# Diagnostic pitfalls after COVID-19 vaccination in melanoma and breast cancer patients: A case series

**DOI:** 10.1016/j.ijscr.2022.106938

**Published:** 2022-03-12

**Authors:** Ellen de Bock, Kari Trumpi, Karijn P.M. Suijkerbuijk, Menno R. Vriens, Milan C. Richir

**Affiliations:** a Department of Surgery, Cancer Center, University Medical Center Utrecht, Utrecht, the Netherlands; b Department of Medical Oncology, Cancer Center, University Medical Center Utrecht, Utrecht, the Netherlands

**Keywords:** Lymphadenopathy, COVID-19, Melanoma, Breast Cancer, SARS-CoV-2

## Abstract

**Introduction:**

During the current Coronavirus Disease 2019 (COVID-19) pandemic, significant COVID-19 disease-reducing developments have been made, culminating in the COVID-19 vaccines. However, COVID-19 vaccines may complicate oncological staging and follow-up oncological disease course since they may induce the enlargement of lymph nodes. Consequently, this uncertainty may lead to increased distress.

**Presentation of cases:**

This case series describes seven patients diagnosed with melanoma or breast cancer in whom lymphadenopathy was observed on oncology imaging after COVID-19 vaccination. Four of these patients underwent additional diagnostic testing, all without malignant cells on pathological examination or suspected metastasis on imaging. The remaining patients were re-evaluated, and the lymphadenopathy was interpreted as an adverse outcome of the recent COVID-19 vaccination. In addition, four out of seven patients were vaccinated in the ipsilateral arm relative to the tumor. Abnormal lymph nodes could be observed up to sixty-nine days after COVID-19 vaccination.

**Discussion and conclusion:**

These findings indicate that a COVID-19 vaccination may result in possible false-positive oncological imaging findings in melanoma and breast cancer patients. Moreover, it is advised to administer the vaccine in the contralateral arm of the primary tumor, suspected breast abnormalities, or after the oncologic imaging in melanoma and breast cancer patients.

## Highlights

• Melanoma and breast cancer patients had lymphadenopathy after COVID-19 vaccination.
• Recent COVID-19 vaccination may lead to additional diagnostic testing.
• Lymphadenopathy was observed up to ninety-six days after the COVID-19 vaccination.
• COVID-19 vaccination may result in false-positive oncological imaging findings.
• Vaccinate in the contralateral arm of the primary tumor or after oncological imaging

## 1 Introduction

Patients diagnosed with breast cancer or melanoma undergo extensive diagnostic staging according to the eighth edition of the American Joint Committee on Cancer (AJCC) tumor, node, metastasis (TNM) staging system [1], [2]. In both patient groups, imaging plays a pivotal role in staging and often consists of a combination of methods, including ultrasound, magnetic resonance imaging (MRI), computed tomography (CT), and/or fluorodeoxyglucose (FDG) positron emission tomography (PET)-CT, depending on the specific patient and their disease stage [3], [4], [5].

During the current Coronavirus Disease 2019 (COVID-19) pandemic, significant COVID-19 disease-reducing developments have been made, culminating in the COVID-19 vaccines [6]. The effectiveness of the COVID-19 vaccine is accomplished because it elicits an immune response. Consequently, this response may be visible as enlarged lymph nodes [7], making it difficult to determine the staging and oncological disease course.

This case series presents seven patients diagnosed with melanoma or breast cancer in whom lymphadenopathy was observed on oncology imaging after COVID-19 vaccination. These non-consecutive cases were retrospectively evaluated and managed in our academic hospital. This case series has been reported in line with the PROCESS Guidelines and registered on www.researchregistry.com with unique identifying number: researchregistry7713 [8].

## 2 Presentation of cases

**Fig. 1:**
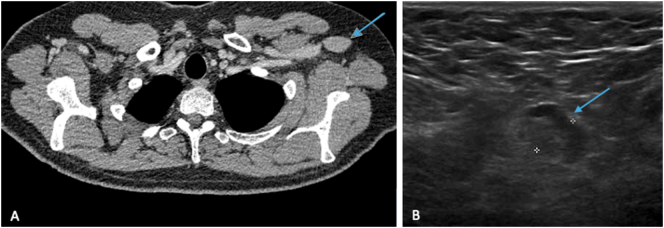
(A) CT-scan showed left axillary lymphadenopathy (arrow), two days after second COVID-19 vaccination. (B) Ultrasound showed decreased left axillary lymph node size compared to the CT-scan (arrow), six days after second COVID-19 vaccination.

A forty-one-year-old man diagnosed with melanoma pT2aN1a, stage IIIB (TNM AJCC), located on the right flank. To check for local recurrence or metastases after recent immunotherapy, a CT scan was performed. He received his second Moderna® COVID-19 vaccination in his left arm two days prior to oncological imaging. The CT showed left axillary lymphadenopathy, suspected for metastases in aspect and size (Fig. 1A). It was then decided to perform an ultrasound-guided biopsy of the corresponding lymph node (Fig. 1B). Pathology examination of the lymph node biopsy showed no signs of malignant cells. Based on this result, routine follow-up was advised.

**Fig. 2:**
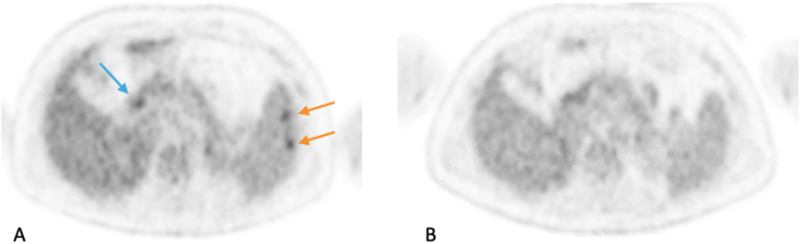
(A) PET-CT showed metabolically active right paracaval lymph nodes (blue arrow) and spleen foci (orange arrows), seventeen days after COVID-19 vaccination. (B) PET-CT showed normalization of previous lymph node and spleen activity, twenty-four days after second COVID-19 vaccination.

A thirty-five-year-old man diagnosed with melanoma pT2bN1a, stage IIIB (TNM AJCC), located on the medial dorsal scapula. To check for local recurrence or metastases during immunotherapy treatment, he underwent a PET-CT. He received his first BioNTech/Pfizer® COVID-19 vaccination seventeen days prior to oncological imaging in his right arm. The FDP-PET revealed, besides new foci of FDG activity in the spleen, metabolically active right paracaval lymph nodes (Fig. 2A). It was advised to repeat the PET-CT within six weeks if no abnormalities were found on the ultrasound. The ultrasound showed no suspicion of metastasis. The PET-CT seven weeks later showed normalization of previously active lymph nodes (Fig. 2B).

**Fig. 3:**
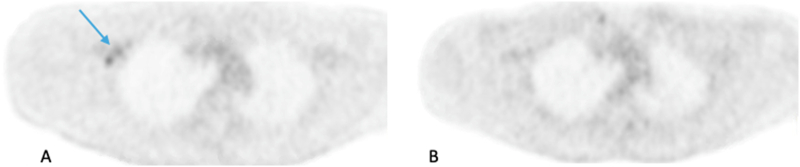
(A) PET-CT showed unenlarged, hypermetabolic lymph node in the right axilla (arrow), thirty-five days after first COVID-19 vaccination. (B) PET-CT showed normalization of previous lymph node activity, seventy-one days after second COVID-19 vaccination.

A forty-three-year-old woman with recurrent melanoma pT1bN1c, stage IIIB (TNM AJCC), located on the right upper arm, underwent a PET-CT to evaluate possible local recurrence or metastases during immunotherapy treatment. She received her first BioNTech/Pfizer® COVID-19 vaccination in her right arm thirty-five days prior to oncological imaging. The PET-CT showed an unenlarged, hypermetabolic lymph node in the right axilla (Fig. 3A). After re-evaluation of the PET-CT at the Multi-disciplinary Team (MDT), there was no convincing evidence of recurrence or metastases. Therefore, it was decided to follow up the patients' disease course with PET-CT. The following PET-CT, after twelve weeks, showed normalization of the previously active lymph nodes (Fig. 3B).

**Fig. 4:**
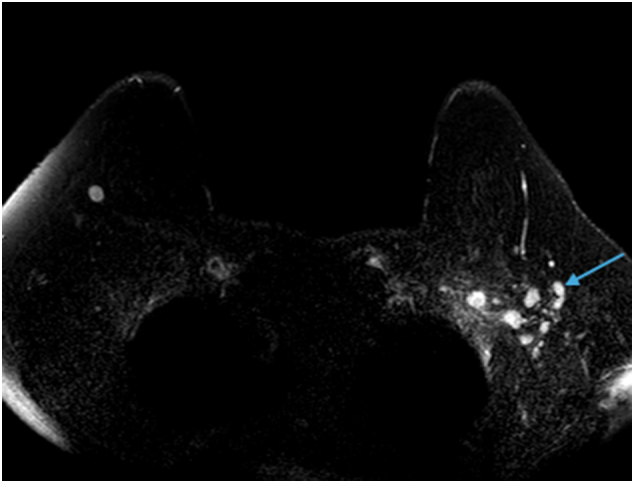
MRI showed left axillary lymph nodes (arrow), twenty-nine days after second COVID-19 vaccination.

A sixty-three-year-old woman diagnosed with right-sided invasive breast carcinoma pT1cN1a(sn) (TNM), ‘no special type’ (NST), Bloom Richardson (BR) grade 2 underwent a MRI to evaluate her oncological status after three cycles of chemotherapy. She received the second dose of the Moderna® COVID-19 vaccination in her left arm twenty-nine days prior to oncological imaging. The MRI showed enlarged left axillary lymph nodes (Fig. 4). At the MDT, the lymphadenopathy was interpreted as an adverse result of the recent COVID-19 vaccination since the lymphadenopathy was located contralateral to the patients' breast cancer. Therefore, it was decided to perform an ultrasound of the lymph nodes if they remain enlarged on a possible future MRI during routine workup. To this date, no additional MRI has been performed.

**Fig. 5:**
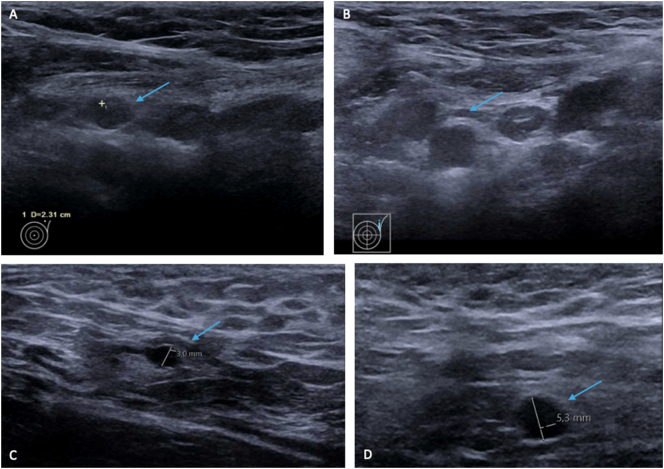
(A) Ultrasound showed four prominent left axillary lymph nodes (arrow), twenty-seven days after COVID-19 vaccination. (B) Ultrasound impressed consistent with previous ultrasound (arrow), ninety-six days after COVID-19 vaccination. (C) Ultrasound showed a right axillary solitary lymph node (arrow), nine days after COVID-19 vaccination. (D) Ultrasound showed multiple prominent left axillary lymph nodes (arrow), four days after COVID-19 vaccination.

A sixty-three-year-old woman diagnosed with previous right-sided breast carcinoma pT1N0M0 and left-sided ductal carcinoma in situ (DCIS), (TNM) BR grade 2 and lung carcinoma underwent a PET-CT for diagnostic imaging of lung carcinoma, showing four FDG-avid left axillary lymph nodes. In addition, the patient received her first AstraZeneca® COVID-19 vaccination twenty-seven days prior to diagnostic imaging in her left arm. The ultrasound of the axilla showed four prominent left axillary lymph nodes with normal morphology (Fig. 5A). It was decided to repeat the ultrasound and compare it with another hospital's previous PET-CT. The additional ultrasound, performed after two months and corresponding with ninety-six days after vaccination, impressed in accordance with the PET-CT and the performed ultrasound (Fig. 5B). It was decided to perform fine-needle aspiration of the most prominent lymph node to ensure no breast cancer metastasis was present. Pathology examination showed no signs of malignant cells. The patient's lung cancer treatment was continued in the primary hospital, and it was advised to follow up the lymph nodes using PET-CT. The CT performed seven months after vaccination showed normalization of the previously active lymph nodes.

A thirty-seven-year-old woman with right-sided invasive breast carcinoma pT1cN0(i-)(sn) (TNM) NST, BR 3 underwent an ultrasound of the right axilla after a recent diagnosis of breast carcinoma to evaluate possible lymphadenopathy. She received her first BioNTech/Pfizer® COVID-19 vaccination nine days prior to oncological imaging in her right arm. The ultrasound of the axilla showed a solitary lymph node in the right axilla with an asymmetrically thickened cortex (Fig. 5C). A cytologic puncture was performed twice in the asymmetric bulge. Pathology examination showed no signs of malignant cells. The sentinel node procedure, performed during bilateral mastectomy, showed no malignant cells.

A forty-four-year-old woman with left-sided breast carcinoma pTis(DCIS)N0(i-)(sn) (TNM), grade two, underwent an ultrasound of the left axilla after a recent diagnosis of DCIS. She received her BioNTech/Pfizer® COVID-19 vaccination four days prior to oncological imaging in her left arm. The ultrasound showed multiple prominent ovate-shaped lymph nodes with hyperechoic centers, a homogeneously thickened cortex, and spherical nodes (Fig. 5D). At the MDT, the lymphadenopathy was interpreted as an adverse result of the recent COVID-19 vaccination and the two-times performed left mammary stereotactic biopsy. It was decided not to perform an additional lymph node biopsy. The sentinel node procedure, performed during bilateral mastectomy, showed no malignant cells.

## 3 Discussion

This study describes the contributing role of the COVID-19 vaccination in the imaging process of patients diagnosed with melanoma or breast cancer. Four out of seven patients required additional imaging or diagnostic methods to evaluate lymph node abnormalities. Oncological imaging or pathological examination of these four patients showed no evidence of malignancy. The other three patients were re-evaluated at the MDT, where it was decided that no additional imaging or pathology examination was needed. These findings indicate that a COVID-19 vaccination may result in possible false-positive oncological imaging findings and subsequently may cause unnecessary anxiety.

Four out of seven patients with false-positive lymph node activation were vaccinated in the ipsilateral arm. These findings are in line with a recent recommendation from The European Society of Breast Imaging (EUSOBI), which advised to perform vaccination in the contralateral arm or thigh in patients with a history of breast cancer [9]. Our findings indicate that vaccination in the contralateral arm is preferable in patients diagnosed with melanoma or breast cancer.

Ninety-six days was the longest interval between vaccination and false-positive oncological imaging in this case series, which is significantly longer than the sixteen days described in an earlier study [10]. However, other patients in this case series showed normalized lymph nodes after a shorter interval, namely twenty-four and seventy-one days. In addition, the EUSOBI advised performing breast imaging at least twelve weeks after the last COVID-19 vaccine dose [9]. Since false-positive lymph nodes in this case series were visible ninety-six days after vaccination, this demonstrates the importance of administering the vaccination in the contralateral arm of the tumor. Allowing lymphadenopathy to be more easily assigned as a result of the COVID-19 vaccination. Further research is warranted to determine the specific time needed after the COVID-19 vaccination to perform oncology imaging to have less false-positive imaging, and subsequent unnecessary distress, in patients diagnosed with melanoma or breast cancer.

This case series has some limitations. First, this study did not investigate whether there were breast cancer or melanoma patients who had recently been vaccinated but did not show lymphadenopathy on imaging. Therefore, an estimate of the probability of false-positive imaging is not possible. Second, only breast cancer and melanoma patients were included in this case series. Consequently, our findings cannot be extrapolated to other types of cancers. However, previous literature describes increased lymph node uptake, most likely due to recent COVID-19 vaccination, in patients diagnosed with neuroendocrine and colorectal malignancy [11].

## 4 Conclusion

A recent COVID-19 vaccination may complicate the diagnostic process of patients with melanoma and breast cancer, leading to additional diagnostic testing, which is associated with increased distress. Hypermetabolic lymph nodes could be observed up to ninety-six days after the COVID-19 vaccination. Therefore, emphasis should be placed on administering the vaccine in the contralateral arm of the primary tumor, suspected breast abnormalities, or after oncological imaging.

## Provenance and peer review

Not commissioned, externally peer-reviewed.

## Informed consent

Written informed consent was obtained from the patients for publication of this case series and accompanying images. A copy of the written consent is available for review by the Editor-in-Chief of this journal on request.

## Ethical approval

The study is exempt from ethical approval in our institution.

## Funding

This research received no specific grant from any funding agency in the public, commercial, or not-for-profit sectors.

## Guarantor

Dr. M.C. Richir.

## Research registration number

Registered on www.researchregistry.com with unique identifying number: researchregistry7713

## CRediT authorship contribution statement

E. de Bock: conceptualization, methodology, data curation, visualization, project administration, writing – original draft, writing – review and editing.
K. Trumpi: conceptualization, methodology, data curation, visualization, supervision, validation, writing – original draft, writing – review & editing.
K.P.M. Suijkerbuijk: conceptualization, methodology, visualization, supervision, validation, writing – original draft, writing – review & editing.
M.R. Vriens: conceptualization, methodology, visualization, supervision, validation, writing – original draft, writing – review & editing.
M.C. Richir: conceptualization, methodology, visualization, supervision, validation, writing – original draft, writing – review & editing.

## Declaration of competing interest

The authors declare that they have no conflict of interest.
